# Magnetic resonance imaging signatures of neuroinflammation in major depressive disorder with religious and spiritual problems

**DOI:** 10.1038/s41598-025-89581-1

**Published:** 2025-02-13

**Authors:** Alexandra Kaszás, Oguz Kelemen, Szabolcs Kéri

**Affiliations:** 1 Department of Cognitive Science, Budapest University of Technology and Economics, Budapest, 1111 Hungary; 2 Department of Behavioral Sciences, Albert Szent-Györgyi Medical School, University of Szeged, Szeged, 6722 Hungary; 3 Department of Psychiatry, Bács-Kiskun County Hospital, Kecskemét, 6000 Hungary; 4 University of Tokaj, Sárospatak College, Sztárai Institute, Sárospatak, 3944 Hungary; 5 Department of Physiology, Albert Szent-Györgyi Medical School, University of Szeged, Szeged, 6720 Hungary

**Keywords:** Human behaviour, Depression, Depression, Social behaviour, Chronic inflammation

## Abstract

Religious and spiritual (R/S) struggles, such as questioning of faith, existential and ethical concerns, and interpersonal conflicts, are associated with depressive symptoms. Neuroinflammation is critical in major depressive disorder (MDD) and is linked to stress associated with R/S problems. This study aimed to investigate whether the presence of DSM-5 R/S problems contributes to neuroinflammation. We recruited 93 MDD patients and 93 healthy controls with and without R/S problems. MRI-based restricted fraction (RF) values, an index of neuroinflammation, were measured in the hippocampus, amygdala, and neocortex. Depression and anxiety were assessed using the Hamilton Depression and Anxiety Rating Scales (HAM-D, HAM-A), while R/S problems were quantified using the Religious and Spiritual Struggles Scale (RSS-14). Results revealed elevated RF values in the amygdala and hippocampus of healthy individuals and MDD patients with R/S problems relative to those without R/S problems, with the highest values in MDD patients with R/S problems. Importantly, R/S problems and depressive symptoms were independent predictors of RF values in the amygdala and hippocampus but not in the cortex. Elevated cortical RF values were associated with MDD. These findings indicate that R/S struggles are not secondary manifestations of depression but may independently contribute to neurobiological changes.

**Supplementary Information:**

The online version contains supplementary material available at 10.1038/s41598-025-89581-1.

## Introduction

There is a significant link between religious/spiritual (R/S) factors and major depressive disorder (MDD), with both positive and negative influences on mental health outcomes. Negative religious coping, such as blaming God or experiencing a loss of faith, is strongly correlated with higher depressive symptoms^1–5^. Adolescents who reported a loss of faith showed less improvement in their depressive symptoms over time^6^. In an international sample of 8,318 volunteers, 10.5% of spiritual participants experienced a depressive episode in the year following the baseline assessment compared to 10.3% of religious participants and 7.0% of secular individuals. However, the findings were heterogeneous, and the difference was significant only in the UK sample^7^.

However, Kasen et al. (2011) observed that religious service attendance was associated with reduced odds of mood disorders, particularly among high-risk individuals exposed to adverse life events^8^. A prominent subjective importance of R/S may provide a protective effect against the recurrence of the disorder, especially in individuals with a family history of depression^9^. The protective effect of R/S has neurobiological underpinnings (a thicker cortex associated with the importance of religion)^10^. McClintock et al. (2019) identified a multidimensional structure of R/S that remained consistent across familial risk groups and diagnostic histories^11^. They noted complex interactions between depression, family risk, and R/S. Specifically, a prior diagnosis of MDD was linked to a weaker R/S commitment in high-risk individuals, heightened contemplation in low-risk individuals, and diminished importance of religion in both high- and low-risk groups^11^. Blazer (2012) reviewed the empirical studies and emphasized the need for longitudinal research to better understand the relationship between R/S and depression^12^. Overall, the evidence suggests that the impact of R/S on depression risk may vary depending on individual factors, cultural context, and specific aspects of R/S engagement.

Positive religious practices, such as forgiveness, connectedness, and finding existential meaning, can help mitigate depressive symptoms by providing social support and a productive coping framework^13,14^. Individuals with stronger spiritual beliefs exhibit better existential well-being, which can buffer against depressive symptoms with critical developmental implications. Adolescence is a crucial stage for spiritual growth, and this developmental period also presents an increased risk of depression, substance misuse, and risk-taking behavior. The timing of spiritual development and the onset of depression suggest a shared biological or developmental process, with positive spiritual coping serving as a protective factor against depression and substance-related issues^15^. However, when spirituality is accompanied by struggles, it can intensify psychological distress, and conversely, stressful life events worsen spiritual struggles^16–21^. Struggles with religious beliefs, such as divine or moral doubts, are linked to lower well-being and higher levels of depression^22^. As discussed above, the effect of R/S on depression varies by cultural context. For example, in the UK, marked spiritual beliefs were associated with an increased likelihood of depression over time^7^.

Recently, studies showed that R/S problems are associated with an exaggerated stress response, abnormal brain activation, and subtle neuroinflammatory changes in limbic areas^23–25^. It is especially relevant for understanding the connection between R/S problems and MDD since neuroinflammation has emerged as a potential key factor in psychiatric disorders. Increasing evidence suggests that immune-mediated processes, such as microglial activation and the release of pro-inflammatory cytokines, play a significant role in the emotional, cognitive, and behavioral symptoms of MDD^26–29^. Elevated levels of pro-inflammatory cytokines, such as interleukin-1 (IL-1), interleukin-6 (IL-6), and tumor necrosis factor-alpha (TNF-α), have been documented in both peripheral blood and cerebrospinal fluid in MDD^30^. Furthermore, post-mortem studies have demonstrated increased expression of inflammation-related genes in the prefrontal cortex and hippocampus of MDD patients^31,32^.

Studies using positron emission tomography (PET) imaging have demonstrated increased microglial activation or cell density in brain regions such as the anterior cingulate cortex, prefrontal cortex, and hippocampus in MDD patients^33^. Translocator protein (18 kDa TSPO) binding, a putative marker of neuroinflammation and microglia density, is higher in MDD in brain regions involved in emotion regulation and cognitive control, including the anterior cingulate cortex, prefrontal cortex, and hippocampus^34–36^. Increased TSPO binding in frontal and cingulate areas is related to attentional dysfunction and suicidal thoughts^37,38^.

Neuroinflammation interacts with key neurobiological correlates of MDD, including serotonin depletion, hippocampal-pituitary-adrenal axis (HPA) dysregulation (abnormal cortisol secretion and stress response), altered microbiota-gut-brain axis, and perturbed hippocampal neurogenesis^39,40^. The kynurenine pathway, a metabolic pathway that converts tryptophan into kynurenine, and HPA axis dysregulation contribute to increased glutamate levels, potentially impacting neurogenesis^41^. Moreover, inflammatory processes may differ among MDD subtypes, with atypical MDD showing distinct inflammatory and neuronal fingerprints^42^. Large-scale studies indicate that lifestyle, brain structure, immunometabolic changes, and genetics contribute to MDD, particularly highlighting the impact of lifestyle (alcohol consumption, diet, physical activity, sleep, smoking, sedentary behavior, and social connectedness)^43^.

From a functional neuroanatomical point of view, the involvement of neuroinflammation in emotional dysregulation is particularly relevant in the amygdala and hippocampus^44^. Studies have found reduced functional connectivity between these regions, the dorsomedial-prefrontal cortex, and the fronto-insular operculum in MDD patients^45^. Structural changes include decreased gray matter volume in the hippocampus and specific cortical areas^46^. Functionally, increased glucose metabolism has been observed in the anterior cingulate cortex^47^. High-resolution connectome analysis revealed decreased connectivity of the hippocampal CA3/4 subregion and reduced clustering of the dentate gyrus in MDD patients. Additionally, dysfunction of a brain network that includes the central nucleus of the amygdala correlates with depression severity^48^. These alterations may contribute to cognitive deficits, particularly in hippocampus-dependent recollection memory, which can persist even after symptom remission^49^.

In a large community sample from the UK biobank, Zhang et al. (2023) evaluated the association between depression, peripheral low-grade inflammation, and neuroinflammation. Zhang et al. (2023) measured C-reactive protein (CRP) levels in the blood plasma, an acute-phase protein produced by the liver during inflammation and stress. The authors also used a specific magnetic resonance imaging (MRI) technique to detect neuroinflammation in the brain (diffusion-basis spectral imaging-based restricted fraction (DBSI-RF) MRI)^50^. The results indicated that elevated peripheral CRP levels were associated with more pronounced depressive symptoms, which were also linked to elevated MRI-restricted fraction (RF) in the amygdala, a putative index of neuroinflammation^50^.

Using Zhang et al.‘s protocol (2023), we also found elevated MRI RF values in the amygdala and hippocampus of individuals with R/S problems, which were associated with subclinical depressive symptoms and peripheral inflammation^25^. However, individuals with R/S problems in this study did not meet the clinical criteria of lifetime or current MDD. Therefore, the interaction between R/S problems and MDD regarding MRI neuroinflammatory markers is unclear.

To elucidate this issue, we investigated four groups: healthy individuals with and without R/S problems and MDD patients with and without R/S problems. We had the following hypotheses driven by previous results discussed above^25,50^: (1) healthy individuals with R/S problems exhibit elevated RF values in the hippocampus and amygdala compared to healthy individuals without R/S problems as a replication of our previous results^25^; (2) MDD patients with and without R/S problems show elevated hippocampal and amygdala RF values relative to healthy individuals; (3) MDD patients with R/S problems display higher depression scores and RF values than MDD patients without R/S problems. Our hypothesis proposed the following order of neuroinflammation severity in the hippocampus and amygdala: healthy individuals with R/S problems < MDD patients without R/S problems < MDD patients with RS problems.

## Methods

### Participants

Patients with MDD (*n* = 93) were recruited from twelve outpatient psychiatric centers and primary care services at the National Psychiatric Center in Budapest and the University of Szeged in Szeged, Hungary. The inclusion criteria were as follows: fulfilling the Diagnostic and Statistical Manual of Mental Disorders (DSM-5) criteria of MDD; age between 18 and 65 years; no significant lifetime psychiatric comorbidities (e.g., evidence of bipolar disorder, psychotic disorders, neurodevelopmental disorders, and substance misuse); never receiving psychotropic medications before the study. The exclusion criteria included neurological disorders or head injury, general medical conditions affecting the central nervous system, substance use within the past six months, pregnancy or breastfeeding, using anti-inflammatory drugs within the past six months, and any general contraindications to MRI.

We also enrolled 93 control participants without a prior history of mental disorders who were matched to the patients with MDD for age, sex, education, and potential confounding variables affecting inflammation (nicotine, caffeine, and alcohol intake, contraception use, body mass index (BMI), chronic diseases, and working night shifts)^51^ (Table 1). We used social media advertisements to recruit control participants with demographic characteristics similar to those of patients with MDD. All participants were Caucasian and declared themselves cisgender (he/him and she/her).

**Table 1:** Demographic and clinical characteristics of the participants.

	Controls	Control with *R*/S problems	MDD	MDD with *R*/S problems	*p*
N	50	43	56	37	–
Age (years)	32.5 ± 12.3	37.9 ± 12.6	38.9 ± 17.1	42.4 ± 15.2	0.02
Education (years)	11.7 ± 3.5	11.6 ± 3.4	11.5 ± 3.5	11.4 ± 3.6	0.9
Sex (male/female)	22/28	19/24	26/30	12/25	> 0.5
Religious affiliation	25 Catholic 17 Protestant 3 Charismatic Christian 3 Judaism 2 Buddhist	28 Catholic 10 Protestant 3 Charismatic Christian 1 Judaism 1 Buddhist	30 Catholic 20 Protestant 2 Charismatic Christian 3 Judaism 1 Judaism-Christian	19 Catholic 11 Protestant 4 Charismatic Christian 3 Judaism	–
BMI	24.5 ± 8.1	24.0 ± 7.0	24.3 ± 7.1	24.1 ± 6.7	0.9
HAM-D	–	–	24.9 ± 7.2	26.8 ± 8.3	0.26
HAM-A	–	–	24.4 ± 11.3	27.0 ± 11.4	0.28
QLDS	–	–	19.1 ± 8.3	20.2 ± 7.9	0.51
RSS-14	22.2 ± 4.8	56.4 ± 9.5	24.0 ± 6.2	51.9 ± 13.0	< 0.001

*MDD* major depressive disorder, *R/S* religious and spiritual, *BMI* body mass index, *HAM-D* Hamilton Depression Rating Scale, *HAM-A* Hamilton Anxiety Rating Scale, *QLDS* Quality of Life in Depression Scale, *RSS-14* Religious and Spiritual Struggles Scale. The p-values are from one-way ANOVAs except for sex where multiple chi-square tests were used.

The study was conducted following the Declaration of Helsinki and approved by the United Ethical Review Committee for Research in Psychology (EPKEB, 2016/032) at the Budapest University of Technology and Economics and the National Medical Research Council (ETT-TUKEB 18814, Budapest, Hungary). Written informed consent was obtained from all subjects.

### Clinical evaluation of diagnosis and symptoms

The patients with MDD and the control volunteers received the structured clinical interview for DSM-5, administered by a trained assessor blind to the study’s aim^52^. Depressive and anxiety symptoms were assessed with the Hamilton Depression Rating Scale (HAM-D) and the Hamilton Anxiety Rating Scale (HAM-A) using semi-structured clinical interviews^53–57^. We also used a self-report scale to assess quality of life.

The HAM-A consists of 14 items, each rated from 0 (not present) to 4 (severe) (total score: 0–56). The scale evaluates both psychic anxiety (mental and psychological distress) and somatic anxiety (physical symptoms). Scoring is categorized into severity ranges: 0–17 indicates mild anxiety, 18–24 indicates moderate anxiety, and 25–30 indicates severe anxiety^53^.

The HAM-D consists of 17 items, each evaluating symptoms such as mood, guilt, suicide ideation, insomnia, anxiety, and weight loss. Each item is scored on a scale of 0 to 2 or 0 to 4, depending on the question, leading to a total score that ranges from 0 to 52. The severity is typically categorized as follows: 0–7: normal or no depression; 8–13: mild depression; 14–18: moderate depression; 19–22: severe depression; > 23: very severe depression^56^.

The Quality of Life in Depression Scale (QLDS) is a patient-reported questionnaire comprising 34 yes-or-no items. Each “Yes” response scores one point, indicating the presence of a problem related to depression, resulting in a total score ranging from 0 to 34; higher scores signify a lower quality of life. The QLDS evaluates critical domains such as emotional well-being, daily activities, social relationships, physical functioning, cognitive functioning, and self-perception. The scale has convincing psychometric properties (Cronbach’s alpha > 0.8)^58,59^.

### Assessing R/S problems

First, to detect R/S problems, we used the structured clinical interview for DSM-5^52^ and the Cultural Formulation Interview^60^. Forty-three control participants and 37 patients with MDD meet the DSM-5 definition of “Problems related to other psychosocial, personal, and environmental circumstances” (Religious or Spiritual Problem, [code: V62.89]^60^. The definition of the R/S problem is the following: “This category can be used when the focus of clinical attention is a religious or spiritual problem. Examples include distressing experiences that involve loss or questioning of faith, problems associated with conversion to a new faith, or questioning of spiritual values that may not necessarily be related to an organized church or religious institution.”^60^.

To quantify R/S problems, we used the Religious and Spiritual Struggles Scale (RSS-14), a 14-item self-report questionnaire^61,62^. This scale assesses six domains of struggles: divine (feelings of anger or disappointment toward a higher power), demonic (experiences of feeling attacked or oppressed by evil forces), interpersonal (conflicts or tensions with others regarding religious or spiritual matters), moral (internal conflicts about right and wrong or ethical dilemmas), doubt (uncertainty or skepticism about one’s religious or spiritual beliefs), and ultimate meaning (existential concerns about the purpose and meaning of life). Each item is rated on a 5-point Likert scale ranging from 1 (“not at all”) to 5 (“a great deal”), resulting in total scores that range from 14 to 70, with higher scores indicating greater levels of struggle. The Hungarian version of the full scale exhibited high internal consistency, with a Cronbach’s alpha of 0.86. However, confirmatory factor analyses have not uniformly supported the six-factor structure in three independent Hungarian samples, and therefore, we used the total scores in the present study.

### Magnetic resonance imaging (MRI)

We acquired diffusion-weighted imaging (DWI) and T1-weighted structural MRI according to the United Kingdom (UK) biobank protocol and our previous study^25,50,63,64^. We used FreeSurfer v7.4.1 for image processing^65^. The setup and the technical parameters were as follows: Philips Achieva 3T scanner, MPRAGE (magnetization-prepared rapid acquisition gradient echo), 3D sagittal acquisition, FOV (square field of view) = 5256 mm, 1 × 1 × 1 mm^3^, TI = 5900 ms, TE (shortest) = 3.16, flip angle: 9 degrees, no fat suppression, full k space, no averages, acquisition time: 6 min and 50 s, acceleration factor: 2. We used a multi-shell approach for DWI (b1 = 1000 s/mm^2^, b2 = 2000 s/mm^2^, 2 × 2 × 2 mm^3^, 50 diffusion encoding directions for each shell). To preprocess the DWI data, we used eddy currents and head motion corrections, outlier slice correction, and gradient distortion correction, as recommended by the UK biobank protocol^50,63^. Putative neuroinflammatory changes were characterized by restricted fraction (RF) from DWI data (diffusion-basis spectral imaging-based restricted fraction, DBSI-RF)^66,67^. We investigated two regions of interest (ROIs), the hippocampus and the amygdala, by using FreeSurfer. We extracted DBSI-RF from these ROIs to obtain comparable results across studies^50,68,69^. The left and right RF values were averaged because they showed high lateralized correlations (left-right correlations: *r*s > 0.8)^50^. We also obtained RF for the whole gray matter of the cortex as a control condition for the limbic structures^50,70^.

### Data analysis

We used Spotifre^®^ Data Science Workbench 14.2.0 (Tibco), JASP 0.19.1, and the R-package for statistical analysis. We calculated the sample size with a medium effect size (α = 0.05, power: 0.80).

After checking the normality of data distribution (Kolmogorov-Smirnov test) and homogeneity of variance (Levene’s test) of all variables, we primarily focused on the RF values. We conducted a multivariate analysis of variance (MANOVA) with the group as between-subjects factors (four groups: healthy controls with and without R/S problems and MDD patients with and without R/S problems) and brain regions (amygdala, hippocampus, and cortex). *F*-tests were used for planned comparisons. The statistical power *(η*^2^*)* was also evaluated in the MANOVA.

To address the relationship between RF values and clinical variables, we conducted hierarchical regression analyses using age, sex, education, BMI, HAM-A, HAM-D, QLDS, and RSS-14 as potential predictors of RF values in the amygdala, hippocampus, and cortex. We also calculated Pearson’s product-moment correlation coefficients with Bonferroni corrections for multiple comparisons. Clinical and demographic variables were compared with one-way ANOVAs, two-tailed *t*-tests, and chi-square tests for categorical variables. The level of uncorrected statistical significance was α < 0.05.

The critical effects obtained with conventional statistics were re-evaluated using a Bayesian data analytic approach. We calculated Bayes Factors (*BF*_*10*_) to characterize the level of evidence by comparing the alternative hypothesis (H_1_) and the null hypothesis (H_0_) (*BF*_*10*_ = 1: no evidence for H_1_; 1 < *BF*_*10*_ < 3: weak evidence for H_1_; 3 < *BF*_*10*_ < 10: moderate evidence for H_1_; *BF*_*10*_ > 10: strong evidence for H_1_).

## Results

### Clinical characteristics and demographics

We first compared the clinical and demographic characteristics of MDD patients and control participants with and without R/S problems (Table 1). No significant differences were found between the four groups in education, sex, and BMI (*p*s > 0.5). Regarding age, MDD patients with R/S problems were older than controls without R/S problems (*p* < 0.05), but the other groups did not differ significantly (*p*s > 0.1).

Regarding the degree of R/S problems, both controls and MDD patients with DSM-5 R/S problems scored higher on the RSS14 scale than individuals without R/S problems (one-way ANOVA, main effect of group: *F*(3,182) = 208.89, *p* < 0.001, *η*^*2*^ = 0.78; post-hoc comparisons: *p* < 0.001). However, healthy controls with R/S problems and MDD patients with R/S problems did not differ on the RSS-14 scale (*p* = 0.08), suggesting a similar level of spiritual distress in the healthy and clinical group.

Contrary to the hypothesis, MDD patients with R/S problems did not exhibit significantly more severe depression and anxiety (HAM-D and HAM-A scores) or a worse quality of life (QLDS scores) relative to MDD patients without R/S problems (*p*s > 0.2) (Table 1).

### RF values in patients with MDD and healthy controls

Next, we compared MRI RF values, a putative index of neuroinflammation, in the amygdala, hippocampus, and cortex of MDD patients with and without R/S problems and healthy control participants with and without R/S problems. The MANOVA conducted on the RF values indicated significant main effects of group (*F*(3,182) = 44.10, *η*^*2*^ = 0.42, *p* < 0.001), brain region (*F*(2,364) = 135.30, *η*^*2*^ = 0.43, *p* < 0.001), and a two-way interaction between group and brain region (*F*(6,364) = 3.15, *η*^*2*^ = 0.05, *p* < 0.05).

Planned comparisons indicated elevated RF values in healthy controls with R/S problems compared to those without R/S problems in the amygdala (*F*(1,182) = 8.93, *p* < 0.01, *BF*_*10*_ = 331.3), in the hippocampus (*F*(1,182) = 8.65, *p* < 0.01, *BF*_*10*_ = 204.3), but not in the cortex (*p* = 0.8, *BF*_*10*_ = 0.2).

**Fig. 1:**
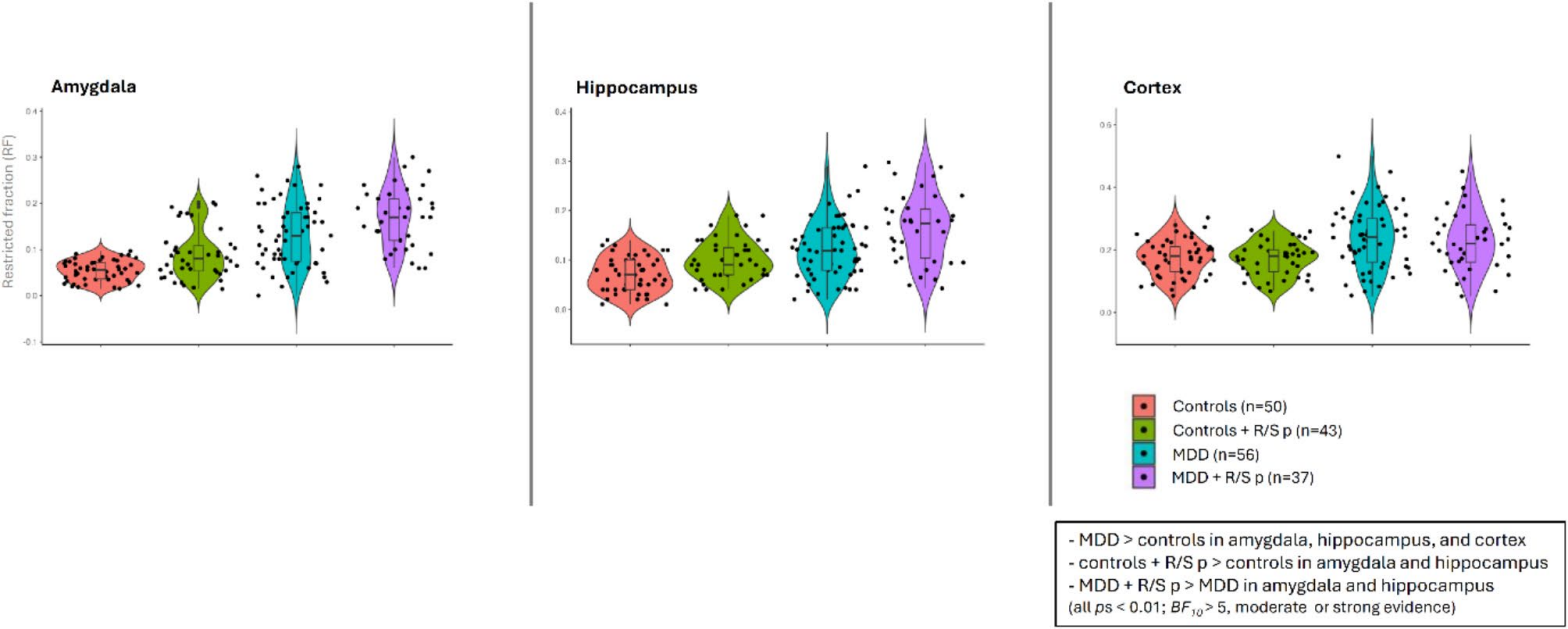
Violin plots and raw data of restricted fraction (RF) values from patients with major depressive disorder (MDD) and healthy controls with and without religious and spiritual problems (R/S p). The horizontal lines indicate the median, the box plots refer to 25 percentiles, and the vertical lines show 50 percentiles.

In the MDD group, patients with R/S problems exhibited higher RF values than patients without R/S problems in the amygdala (*F*(1,182) = 10.76, *p* < 0.002, *BF*_*10*_ = 4.9), in the hippocampus (*F*(1,182) = 15.76, *p* < 0.001, *BF*_*10*_ = 21.1), but not in the cortex (*p* = 0.7, *BF*_*10*_ = 0.2) (Fig. 1; for a detailed descriptive statistics, see supplementary material).

Overall, patients with MDD had elevated RF values relative to controls in the amygdala (*F*(1,182) = 94.80, *p* < 0.001, *BF*_*10*_ = 3.2 × 10^13^), in the hippocampus (*F*(1,182) = 54.46, *p* < 0.001, *BF*_*10*_ = 2.6 × 10^7^), and in the cortex (*F*(1,182) = 26.44, *p* < 0.001, *BF*_*10*_ = 35043.4). Interestingly, healthy controls with R/S problems did not differ from patients with MDD without R/S problems in the hippocampus (*p* = 0.1, *BF*_*10*_ = 0.9) (Fig. 1).

### Clinical and R/S predictors of RF values in MDD

We performed multiple regression analyses to elucidate the clinical predictors of RF values in MDD, controlled for age, education, sex, and BMI.

In the amygdala, there were two significant predictors of RF: HAM-D scores (b* = 0.28, *R*^2^ = 0.27, *t*(84) = 2.56, *p* < 0.05) and RSS-14 scores (b* = 0.22, *R*^2^ = 0.18, *t*(84) = 2.19, *p* < 0.05). The Bayesian regression analysis revealed the highest level of evidence (*BF*_*10*_ = 8.7, *R*^2^ = 0.27) when HAM-D, RSS-14, age, and education were included in the model.

In the hippocampus, we identified three significant predictors: HAM-D scores (b* = 0.50, *R*^2^ = 0.28, *t*(84) = 5.45, *p* < 0.001), HAM-A scores (b* = 0.21, *R*^2^ = 0.13, *t*(84) = 2.44, *p* < 0.05), and RSS-14 scores (b* = 0.19, *R*^2^ = 0.18, *t*(84) = 2.20, *p* < 0.05). The Bayesian regression analysis revealed the highest level of evidence (*BF*_10_ = 30.5, *R*^2^ = 0.46) when HAM-D, HAM-A, RSS-14, and age were included in the model.

In the cortex, there was a single significant predictor: education (b* = − 0.33, *R*^2^ = 0.10, *t*(84) = − 3.14, *p* < 0.05), but the Bayesian approach did not indicate an acceptable level of evidence (*BF*_10_ = 1, *R*^2^ = 0.10).

**Fig. 2:**
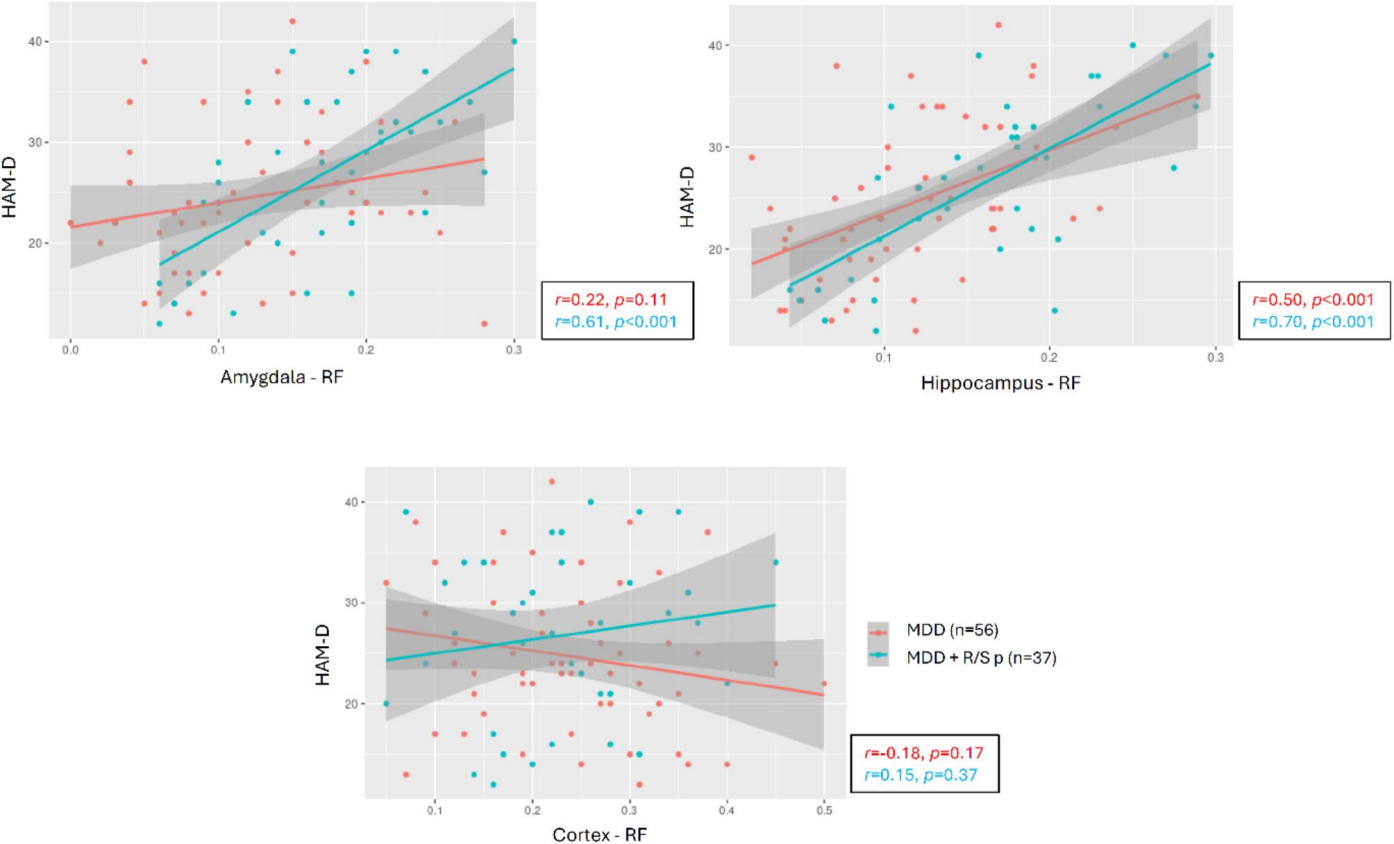
Correlations between depressive symptoms (Hamilton Depression Rating Scale, HAM-D) and restricted fractions (RF) in patients with major depressive disorder (MDD) with and without religious and spiritual problems (R/S p).

**Fig. 3:**
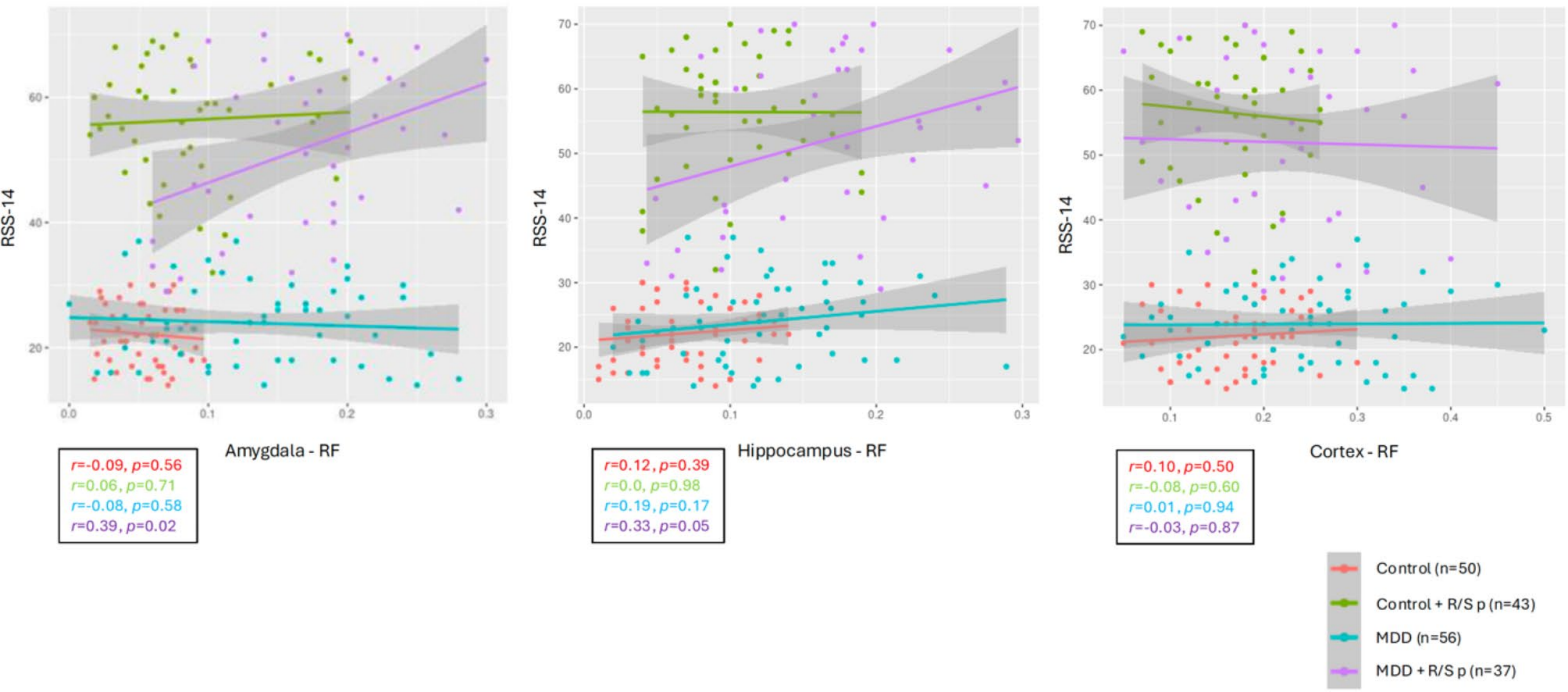
Correlation between religious and spiritual struggles scores (RSS-14) and restricted fractions (RF) in healthy controls and patients with major depressive disorder with and without religious and spiritual problems (R/S p).

Table 2 depicts Pearson’s product-moment correlation coefficients and Bayesian correlations between the RF values and clinical and demographic measures in MDD (the detailed correlation coefficients are shown in the supplementary material). Figures 2 and 3 show the correlations between RF values and their significant clinical predictors in the amygdala and hippocampus in MDD and healthy controls.

**Table 2:** Correlations between MRI markers of neuroinflammation (RF) and clinical and demographic parameters in MDD.

RF	Age	Education	BMI	HAM-D	HAM-A	QLDS	RSS14
Amygdala							
*r*	− 0.18	− 0.28*	0.04	0.39**	0.29*	0.10	0.32*
*BF*_*10*_	0.59	6.0	0.14	242.0	6.0	0.20	14.03
Hippocampus							
*r*	0.18	− 0.14	0.11	0.59**	0.36**	0.13	0.41**
*BF*_*10*_	0.56	0.30	0.22	1.94 × 10^7^	68.22	0.28	441.89
Cortex							
*r*	0.02	− 0.32*	0.12	− 0.05	0.13	− 0.10	− 0.03
*BF*_*10*_	0.13	16.76	0.26	0.14	0.28	0.20	0.14

*RF* restricted fraction, *BMI* body mass index, *HAM-D* Hamilton Depression Rating Scale, *HAM-A* Hamilton Anxiety Rating Scale, *QLDS* Quality of Life in Depression, *RSS-14* Religious and Spiritual Struggle Scale, *r* Pearson’s correlation coefficient, *BF* Bayes factor.

*p < 0.05, **p < 0.002 (Bonferroni-corrected).

#### Predictors of RF values and correlations in the control group

We also assessed whether R/S problems predict RF values in the control group. In the amygdala, the RSS-14 scores significantly predicted the RF values, controlled for age, education, sex, and BMI (b* = 0.37, *R*^2^ = 0.08, *t*(87) = 3.51, *p* < 0.01). A similar effect was found in the hippocampus (b* = 0.36, *R*^2^ = 0.08, *t*(87) = 3.56, *p* < 0.01) but not in the cortex (*p* > 0.5). However, the Bayesian analysis failed to confirm the results from the conventional statistics, indicating no convincing evidence that the RSS-14 scores significantly predicted RF values in the amygdala and hippocampus (*BF*_10_ = 1).

There was a significant positive correlation between amygdala-RF and RSS-14 scores (*r* = 0.38, *p* < 0.01; *BF*_10_ = 142.4) and between hippocampus-RF and RSS-14 scores (*r* = 0.37, *p* < 0.01; *BF*_10_ = 104.3) in the whole sample but no such correlations in the cortex (*r* = − 0.04, *BF*_10_ *=* 0.14). Figure 3 shows the correlations in each group, including MDD.

## Discussion

The present study highlights the role that R/S struggles play in neuroinflammatory processes in individuals with MDD. The findings contribute to a growing body of research that demonstrates the complex interplay between psychological, spiritual, and neurobiological factors in depression^71–73^. Specifically, the study’s key findings—that both healthy individuals with R/S struggles and MDD patients with R/S struggles exhibit elevated MRI-RF values in the amygdala and hippocampus—provide new insights into the underlying mechanisms of MDD, particularly as they relate to religious and spiritual distress.

The results suggest that neuroinflammatory processes in the limbic system, indicated by increased RF values, may worsen when religious and spiritual struggles are also present. The hippocampus and amygdala are critical regions involved in emotion regulation and memory processing, often disrupted in MDD^74–76^, and are also associated with R/S problems. The observation that individuals without a clinical diagnosis of MDD but who experience R/S struggles show neuroinflammatory changes similar to those of MDD patients indicates that R/S struggles may represent a significant risk factor for depression or, at the very least, contribute to the same physiological processes that underlie depressive symptoms. However, it is essential to emphasize that depressive symptoms and R/S problems were independent predictors of RF in the amygdala and hippocampus, demonstrating that R/S issues are not secondary manifestations of depression. MDD patients, regardless of R/S problems, did not differ in the severity of depressive symptoms, anxiety, or quality of life. Additionally, some individuals may experience R/S problems and increased amygdala and hippocampus RF without being clinically diagnosed with MDD. This observation also aligns with data suggesting that positive R/S may provide neuroprotective effects^10^, while negative R/S disrupts brain function similarly to that observed in MDD.

The present findings are consistent with previous research, indicating that inflammation plays a central role in the pathophysiology of MDD^74–76^. Numerous studies investigated fundamental mechanisms of inflammation in MDD, such as elevated levels of pro-inflammatory cytokines and microglial activation. However, developing clinically accessible, non-invasive brain imaging techniques to measure neuroinflammation remains an unresolved issue. Advanced diffusion-weighted MRI approaches used in the present study are among the possible solutions^50^. Previously, in a large-scale population-based survey including biobank data from 11,512 individuals, it has been demonstrated that higher amygdala-RF was associated with elevated depression, especially in those with a lifetime history of depression^50^.

However, associations between global-RF or hippocampus-RF and depression were not significant, and no RF values linked depression with peripheral blood markers of low-grade inflammation^50^. Our results were different because we found increased hippocampus-RF, which was more strongly associated with depressive and even anxiety symptoms than that found in the case of the amygdala. Finally, we also observed increased cortical RF values in MDD, which was the most essential discriminating feature from R/S problems without MDD. However, putative neuroinflammatory changes in the cortex of MDD patients were not linked to depressive or anxiety symptoms, probably indicating a separate cognitive symptom dimension. The differences between the results of the Zhang et al. (2023) data and the present study may be attributed to distinct sample characteristics. In the present study, we included clinically defined MDD patients who never received any treatment before, whereas the Zhang et al. (2023) sample was from an extensive database of heterogeneous individuals^50^.

These findings have two main implications. First, they underscore the importance of considering R/S struggles as a separate psychological factor that may have measurable effects on brain health. Second, the findings suggest that addressing R/S struggles in therapeutic interventions could be beneficial for patients with MDD. For example, integrating spiritual counseling or interventions that promote positive religious coping could potentially mitigate the neuroinflammatory processes observed in these individuals^16^.

The strength of the present study is the application of MRI-based techniques to quantify neuroinflammation, allowing for a direct assessment of physiological changes associated with R/S struggles. The methodological rigor, including the application of the DBSI-RF MRI protocol^50^ and controlling several factors affecting inflammation, ensures that the observed differences in RF values are reliable indicators, adding significant value to the existing neuroimaging literature in MDD. Finally, we included never-treated patients with MDD, which excludes the confounding effects of psychotropic medications.

However, several limitations should be acknowledged. First, the cross-sectional design limits the ability to infer causality and the interaction between MDD, R/S struggles, and neuroinflammation. While the study shows a clear association, it remains unclear whether R/S struggles contribute to increased neuroinflammation or individuals with pre-existing neuroinflammation are more likely to experience R/S struggles. It is also unclear whether neuroinflammatory changes in R/S struggles are reliable predictors of the development of MDD. Longitudinal studies are necessary to clarify this relationship. The available prospective studies examining the relationship between R/S and depression are heterogeneous, and a definitive synthesis is not possible^77^. Half of the published studies reported that positive R/S predicted a modest decrease in depression, but future investigations into the association between R/S struggles and depressive disorders are indispensable^77^.

Second, the study sample is confined to individuals who identify themselves as Caucasian in Eastern Europe, limiting the generalizability to more diverse populations. Religious and spiritual experiences are deeply embedded in cultural contexts, and the way R/S struggles manifest and affect mental health may vary across different cultural and ethnic groups. Future studies should include more diverse and larger samples to examine whether the neuroinflammatory effects of R/S struggles are consistent across various demographic groups^78^. However, convenience sampling and potential biases are difficult to control because of the unique characteristics of the population. Individuals with R/S problems often refuse to participate in psychological and neurobiological investigations because of their religious beliefs, attitudes, and attributions^79^.

While the study provides evidence that R/S struggles are associated with neuroinflammatory changes in the amygdala and hippocampus, the underlying mechanisms driving these changes remain unclear. The stress associated with religious and spiritual conflicts may activate the hypothalamic-pituitary-adrenal (HPA) axis, leading to the release of cortisol and other stress-related hormones^40^. Alternatively, chronic R/S struggles may disrupt sleep patterns, which in turn could exacerbate neuroinflammatory processes^80^. Future research should explore these potential mechanisms to understand how R/S distress translates into neurobiological changes. Finally, we could not separately investigate the dimensions of R/S struggles because of the inconsequent nature of the RSS-14 scale’s factor structure in Hungarian samples.

In conclusion, this study provides evidence that R/S struggles are not only significant psychological stressors but may also contribute to neuroinflammatory processes in the brain, which are associated with more severe alterations in MDD. These findings highlight the importance of addressing spiritual distress in therapeutic settings and pave the way for future research to explore the mechanisms through which this distress influences brain health. Further investigation into the neurobiological underpinnings of R/S struggles, including longitudinal studies and more diverse samples, will be crucial for understanding this complex relationship.

## Electronic supplementary material

Below is the link to the electronic supplementary material.

Supplementary Material 1

Supplementary Material 2

Supplementary Material 3

Supplementary Material 4

Supplementary Material 5

Supplementary Material 6

Supplementary Material 7

Supplementary Material 8

## Data Availability

The datasets generated and analyzed during the present study are available from the corresponding author upon reasonable request.
