# Supplementary material for: Magnetic resonance imaging signatures of neuroinflammation in major depressive disorder with religious and spiritual problems
Source: Sci Rep. 2025 Feb 13;15:5407. doi: 10.1038/s41598-025-89581-1 (PMC11825903; doi:10.1038/s41598-025-89581-1)
Supplement: Supplementary file 1 — Supplementary Material 1 [file 41598_2025_89581_MOESM1_ESM.pdf]

Results

Bayesian Correlation

Correlation matrix from controls with religious and spiritual problems (n=43).

Bayesian Pearson Correlations

Variable		amyg	hippo	cortex	age	edu	BMI	RSS14
1. amyg	Pearson's r	—						
	BF_{10}	—						
2. hippo	Pearson's r	0.265	—					
	BF_{10}	0.794	—					
3. cortex	Pearson's r	−0.042	0.098	—				
	BF_{10}	0.197	0.230	—				
4. age	Pearson's r	0.201	0.196	0.012	—			
	BF_{10}	0.425	0.411	0.191	—			
5. edu	Pearson's r	−0.061	0.347	0.107	0.145	—		
	BF_{10}	0.205	2.332	0.238	0.288	—		
6. BMI	Pearson's r	−0.011	−0.025	−0.233	0.032	−0.060	—	
	BF_{10}	0.190	0.192	0.570	0.194	0.204	—	
7. RSS14	Pearson's r	0.058	−0.003	−0.082	0.109	−0.230	−0.379	—
	BF_{10}	0.203	0.190	0.217	0.240	0.552	3.935	—

Bayesian Correlation - Conclusion: Click here to add text
