# Supplementary material for: Magnetic resonance imaging signatures of neuroinflammation in major depressive disorder with religious and spiritual problems
Source: Sci Rep. 2025 Feb 13;15:5407. doi: 10.1038/s41598-025-89581-1 (PMC11825903; doi:10.1038/s41598-025-89581-1)
Supplement: Supplementary file 2 — Supplementary Material 2 [file 41598_2025_89581_MOESM2_ESM.pdf]

Results

Correlation matrix from controls, including those with and without spiritual and religious problems.

Bayesian Correlation

Bayesian Pearson Correlations

Variable		amyg	hippo	cortex	age	edu	BMI	RSS14
1. amyg	Pearson's r	—						
	BF_{10}	—						
2. hippo	Pearson's r	0.228	—					
	BF_{10}	1.391	—					
3. cortex	Pearson's r	0.017	−0.056	—				
	BF_{10}	0.131	0.149	—				
4. age	Pearson's r	0.203	0.157	0.018	—			
	BF_{10}	0.850	0.393	0.132	—			
5. edu	Pearson's r	−0.113	0.085	−0.019	0.047	—		
	BF_{10}	0.230	0.179	0.132	0.143	—		
6. BMI	Pearson's r	0.004	−0.014	−0.128	−0.002	−0.098	—	
	BF_{10}	0.130	0.131	0.269	0.130	0.200	—	
7. RSS14	Pearson's r	0.381	0.373	−0.037	0.224	−0.040	−0.144	—
	BF_{10}	142.387	104.337	0.138	1.289	0.139	0.331	—
