# Supplementary material for: Magnetic resonance imaging signatures of neuroinflammation in major depressive disorder with religious and spiritual problems
Source: Sci Rep. 2025 Feb 13;15:5407. doi: 10.1038/s41598-025-89581-1 (PMC11825903; doi:10.1038/s41598-025-89581-1)
Supplement: Supplementary file 3 — Supplementary Material 3 [file 41598_2025_89581_MOESM3_ESM.pdf]

Results

Correlation matrix from controls, including only without spiritual and religious problems (n=50).

Bayesian Correlation

Bayesian Pearson Correlations

Variable		amyg	hippo	cortex	age	edu	BMI	RSS14
1. amyg	Pearson's r	—						
	BF_{10}	—						
2. hippo	Pearson's r	−0.307	—					
	BF_{10}	1.746	—					
3. cortex	Pearson's r	0.184	−0.169	—				
	BF_{10}	0.391	0.345	—				
4. age	Pearson's r	0.021	−0.029	0.038	—			
	BF_{10}	0.178	0.180	0.182	—			
5. edu	Pearson's r	−0.275	−0.146	−0.115	−0.035	—		
	BF_{10}	1.083	0.290	0.240	0.181	—		
6. BMI	Pearson's r	0.070	0.015	−0.060	−0.016	−0.127	—	
	BF_{10}	0.197	0.177	0.192	0.177	0.256	—	
7. RSS14	Pearson's r	−0.085	0.124	0.098	0.028	0.108	−0.257	—
	BF_{10}	0.208	0.252	0.220	0.179	0.231	0.853	—
