# Supplementary material for: Magnetic resonance imaging signatures of neuroinflammation in major depressive disorder with religious and spiritual problems
Source: Sci Rep. 2025 Feb 13;15:5407. doi: 10.1038/s41598-025-89581-1 (PMC11825903; doi:10.1038/s41598-025-89581-1)
Supplement: Supplementary file 4 — Supplementary Material 4 [file 41598_2025_89581_MOESM4_ESM.pdf]

Results

Bayesian Correlation

Correlation matrix from patients with major depressive disorder without religious and spiritual problems (n=56). HAM_D, Hamilton Rating Scale for Depression; HAM_A, Hamilton Rating Scale for Anxiety; QLIFED, Quality of Life in Depression; RSS14, Religious and Spiritual Struggle Scale

Bayesian Pearson Correlations

Variable		amyg	hippo	cortex	HAM_D	HAM_A	age	QLIFED	edu	BMI	RSS14
1. amyg	Pearson's r	—									
	BF_{10}	—									
2. hippo	Pearson's r	0.404	—								
	BF_{10}	17.409	—								
3. cortex	Pearson's r	0.019	−0.053	—							
	BF_{10}	0.168	0.179	—							
4. HAM_D	Pearson's r	0.216	0.479	−0.186	—						
	BF_{10}	0.578	149.927	0.418	—						
5. HAM_A	Pearson's r	0.167	0.392	0.055	0.199	—					
	BF_{10}	0.348	12.993	0.180	0.479	—					
6. age	Pearson's r	−0.319	0.208	−0.086	−0.101	0.159	—				
	BF_{10}	2.729	0.529	0.202	0.218	0.325	—				
7. QLIFED	Pearson's r	−0.042	0.107	−0.096	0.284	0.184	−0.138	—			
	BF_{10}	0.175	0.226	0.213	1.494	0.410	0.275	—			
8. edu	Pearson's r	−0.196	−0.041	−0.322	−0.104	−0.153	0.226	−0.244	—		
	BF_{10}	0.464	0.174	2.867	0.221	0.309	0.657	0.824	—		
9. BMI	Pearson's r	0.141	0.281	0.196	0.057	0.129	−0.071	0.038	−0.099	—	
	BF_{10}	0.281	1.428	0.462	0.182	0.257	0.190	0.173	0.216	—	
10. RSS14	Pearson's r	−0.075	0.187	0.010	0.274	0.145	−0.050	0.186	−0.133	0.323	—
	BF_{10}	0.193	0.423	0.167	1.272	0.289	0.178	0.418	0.266	2.929	—

Bayesian Correlation - Conclusion: Click here to add text
