# Supplementary material for: Magnetic resonance imaging signatures of neuroinflammation in major depressive disorder with religious and spiritual problems
Source: Sci Rep. 2025 Feb 13;15:5407. doi: 10.1038/s41598-025-89581-1 (PMC11825903; doi:10.1038/s41598-025-89581-1)
Supplement: Supplementary file 5 — Supplementary Material 5 [file 41598_2025_89581_MOESM5_ESM.pdf]

Results

Correlation matrix from patients with major depressive disorder with spiritual and religious problems (n=37). HAM_D, Hamilton Depression Rating Scale; HAM_A, Hamilton Anxiety Rating Scale; QLIFED, Quality of Life in Depression; RSS14, Religious and Spiritual Struggles Scale

Bayesian Correlation

Bayesian Pearson Correlations

Variable		amyg	hippo	cortex	HAM_D	HAM_A	age	QLIFED	edu	BMI	RSS14
1. amyg	Pearson's r	—									
	BF_{10}	—									
2. hippo	Pearson's r	0.450	—								
	BF_{10}	8.719	—								
3. cortex	Pearson's r	0.028	0.239	—							
	BF_{10}	0.207	0.543	—							
4. HAM_D	Pearson's r	0.619	0.702	0.153	—						
	BF_{10}	631.456	15912.047	0.304	—						
5. HAM_A	Pearson's r	0.435	0.291	0.265	0.312	—					
	BF_{10}	6.667	0.887	0.688	1.120	—					
6. age	Pearson's r	−0.037	0.081	0.226	0.011	−0.292	—				
	BF_{10}	0.209	0.228	0.491	0.205	0.897	—				
7. QLIFED	Pearson's r	0.297	0.123	−0.098	0.272	−0.160	0.312	—			
	BF_{10}	0.950	0.264	0.240	0.734	0.314	1.119	—			
8. edu	Pearson's r	−0.462	−0.270	−0.323	−0.221	−0.450	−0.166	0.050	—		
	BF_{10}	11.028	0.723	1.278	0.471	8.792	0.326	0.213	—		
9. BMI	Pearson's r	−0.121	−0.097	-6.971×10^{-4}	−0.029	−0.128	−0.029	−0.283	0.102	—	
	BF_{10}	0.261	0.240	0.205	0.207	0.269	0.207	0.815	0.243	—	
10. RSS14	Pearson's r	0.389	0.327	−0.029	0.594	0.079	0.152	0.269	−0.250	0.168	—
	BF_{10}	3.117	1.334	0.208	281.128	0.227	0.302	0.711	0.596	0.329	—
