# Supplementary material for: Magnetic resonance imaging signatures of neuroinflammation in major depressive disorder with religious and spiritual problems
Source: Sci Rep. 2025 Feb 13;15:5407. doi: 10.1038/s41598-025-89581-1 (PMC11825903; doi:10.1038/s41598-025-89581-1)
Supplement: Supplementary file 6 — Supplementary Material 6 [file 41598_2025_89581_MOESM6_ESM.pdf]

Results

Correlation matrix from the patients with major depressive disorder, including those with and without teligious and spiritual problems. HAM_D, Hamilton Depression Rating Scale; HAM_A, Hamilton Anxiety Rating Scale; QLIFED, Quality of Life in Depression; RSS14, Religious and Spiritual Struggles Scale-14

Bayesian Correlation

Bayesian Pearson Correlations

Variable		amyg	hippo	cortex	HAM_D	HAM_A	age	QLIFED	BMI	edu	RSS14
1. amyg	Pearson's r	—									
	BF_{10}	—									
2. hippo	Pearson's r	0.470	—								
	BF_{10}	8871.722	—								
3. cortex	Pearson's r	0.012	0.055	—							
	BF_{10}	0.130	0.149	—							
4. HAM_D	Pearson's r	0.394	0.587	−0.048	—						
	BF_{10}	241.951	$1.937 \times 10^{+7}$	0.144	—						
5. HAM_A	Pearson's r	0.287	0.362	0.131	0.257	—					
	BF_{10}	5.990	68.215	0.281	2.745	—					
6. age	Pearson's r	−0.183	0.180	0.021	−0.043	0.004	—				
	BF_{10}	0.589	0.560	0.132	0.141	0.130	—				
7. QLIFED	Pearson's r	0.097	0.129	−0.099	0.283	0.059	0.029	—			
	BF_{10}	0.197	0.275	0.201	5.383	0.151	0.134	—			
8. BMI	Pearson's r	0.040	0.109	0.124	0.019	0.028	−0.057	−0.081	—		
	BF_{10}	0.139	0.220	0.258	0.132	0.134	0.150	0.174	—		
9. edu	Pearson's r	−0.287	−0.136	−0.322	−0.154	−0.272	0.079	−0.129	−0.021	—	
	BF_{10}	6.039	0.297	16.764	0.378	3.974	0.171	0.273	0.132	—	
10. RSS14	Pearson's r	0.316	0.409	−0.034	0.350	0.149	0.118	0.178	0.110	−0.110	—
	BF_{10}	14.031	441.889	0.137	44.461	0.352	0.242	0.548	0.222	0.223	—
