# Supplementary material for: Magnetic resonance imaging signatures of neuroinflammation in major depressive disorder with religious and spiritual problems
Source: Sci Rep. 2025 Feb 13;15:5407. doi: 10.1038/s41598-025-89581-1 (PMC11825903; doi:10.1038/s41598-025-89581-1)
Supplement: Supplementary file 7 — Supplementary Material 7 [file 41598_2025_89581_MOESM7_ESM.pdf]

Results

Descriptive Statistics

Detailed descriptive statistics in the control group with no religious and spiritual problems (0) and those with religious and spiritual problems (1). Amyg (amygdala), hippo (hippocampus), and cortex refer to restricted fraction values. RSS14 - Religious and Spiritual Struggles Scale-14.

Descriptive Statistics

	amyg		cortex		age		hippo		edu		BMI		RSS14	
	0	1	0	1	0	1	0	1	0	1	0	1	0	1
Valid	50	43	50	43	50	43	50	43	50	43	50	43	50	43
Missing	0	0	0	0	0	0	0	0	0	0	0	0	0	0
Median	0.057	0.081	0.180	0.180	31.000	37.000	0.070	0.090	12.000	12.000	23.000	23.000	22.000	58.000
Mean	0.055	0.089	0.174	0.170	32.520	37.884	0.070	0.102	11.640	11.628	24.460	24.023	22.160	56.419
Std. Error of Mean	0.003	0.008	0.008	0.008	1.742	1.920	0.005	0.006	0.502	0.532	1.152	1.059	0.668	1.443
95% CI Mean Upper	0.061	0.105	0.190	0.186	36.022	41.758	0.080	0.114	12.649	12.702	26.775	26.161	23.503	59.331
95% CI Mean Lower	0.048	0.073	0.157	0.153	29.018	34.010	0.060	0.090	10.631	10.553	22.145	21.885	20.817	53.506
Std. Deviation	0.022	0.052	0.059	0.053	12.321	12.587	0.035	0.039	3.550	3.492	8.145	6.947	4.727	9.465
Skewness	−0.081	0.808	−0.068	−0.181	0.630	0.552	0.144	0.481	0.654	0.570	0.750	0.558	0.029	−0.639
Std. Error of Skewness	0.337	0.361	0.337	0.361	0.337	0.361	0.337	0.361	0.337	0.361	0.337	0.361	0.337	0.361
Kurtosis	−1.007	−0.205	−0.601	−0.755	−0.384	0.091	−0.993	−0.292	−0.637	−0.842	−0.159	−0.405	−1.140	−0.200
Std. Error of Kurtosis	0.662	0.709	0.662	0.709	0.662	0.709	0.662	0.709	0.662	0.709	0.662	0.709	0.662	0.709
Minimum	0.015	0.015	0.050	0.070	18.000	18.000	0.010	0.040	8.000	8.000	12.000	11.000	14.000	32.000
Maximum	0.097	0.202	0.300	0.260	65.000	65.000	0.140	0.190	20.000	19.000	46.000	40.000	30.000	70.000
25th percentile	0.036	0.053	0.130	0.130	21.000	29.000	0.040	0.070	8.000	8.000	18.000	19.000	18.000	50.500
50th percentile	0.057	0.081	0.180	0.180	31.000	37.000	0.070	0.090	12.000	12.000	23.000	23.000	22.000	58.000
75th percentile	0.072	0.108	0.210	0.200	42.000	44.000	0.100	0.125	13.750	13.500	29.750	28.500	26.000	64.000

Descriptive Statistics - Conclusion: Click here to add text
