# Supplementary material for: Magnetic resonance imaging signatures of neuroinflammation in major depressive disorder with religious and spiritual problems
Source: Sci Rep. 2025 Feb 13;15:5407. doi: 10.1038/s41598-025-89581-1 (PMC11825903; doi:10.1038/s41598-025-89581-1)
Supplement: Supplementary file 8 — Supplementary Material 8 [file 41598_2025_89581_MOESM8_ESM.pdf]

Results

Descriptive Statistics

Detailed descriptive statistics from the group of patients with major depressive disorder (MDD) including those without religious and spiritual problems (2) and those with religious and spiritual problmes (3). Amyg (amygdala), hippo (hippocampus), and cortex refer to restricted fraction values. HAM_D, Hamilton Rating Scale for Depression, HAM_A, Hamilton Rating Scale for Anxiety, QLIFED, Quality of Life in Depression, RSS14, Religious and Spiritual Struggle Scale

Descriptive Statistics

	amyg		hippo		cortex		HAM_D		HAM_A		age		QLIFED		edu		BMI		RSS14	
	2	3	2	3	2	3	2	3	2	3	2	3	2	3	2	3	2	3	2	3
Valid	56	37	56	37	56	37	56	37	56	37	56	37	56	37	56	37	56	37	56	37
Missing	0	0	0	0	0	0	0	0	0	0	0	0	0	0	0	0	0	0	0	0
Median	0.130	0.170	0.119	0.174	0.235	0.220	24.000	28.000	24.000	27.000	34.500	44.000	18.000	19.000	12.000	12.000	22.500	22.000	24.000	54.000
Mean	0.132	0.170	0.120	0.163	0.236	0.229	24.911	26.757	24.375	26.973	38.911	42.405	19.054	20.189	11.482	11.459	24.339	24.108	23.946	51.919
Std. Error of Mean	0.009	0.010	0.008	0.011	0.013	0.015	0.966	1.369	1.509	1.877	2.289	2.493	1.114	1.301	0.469	0.595	0.944	1.094	0.829	2.137
95% CI Mean Upper	0.150	0.191	0.136	0.186	0.262	0.260	26.847	29.533	27.399	30.781	43.498	47.461	21.286	22.828	12.422	12.666	26.231	26.326	25.609	56.253
95% CI Mean Lower	0.113	0.149	0.105	0.141	0.209	0.198	22.975	23.980	21.351	23.165	34.324	37.350	16.821	17.550	10.542	10.253	22.448	21.890	22.284	47.585
Std. Deviation	0.069	0.064	0.058	0.068	0.099	0.094	7.229	8.328	11.292	11.420	17.128	15.163	8.335	7.916	3.511	3.618	7.064	6.653	6.207	12.999
Skewness	0.191	−0.005	0.506	0.097	0.294	0.255	0.306	−0.176	0.308	−0.233	0.424	−0.184	0.395	0.390	0.674	0.774	0.875	0.943	0.241	−0.222
Std. Error of Skewness	0.319	0.388	0.319	0.388	0.319	0.388	0.319	0.388	0.319	0.388	0.319	0.388	0.319	0.388	0.319	0.388	0.319	0.388	0.319	0.388
Kurtosis	−0.851	−0.724	0.196	−0.663	−0.194	−0.278	−0.514	−1.068	−0.506	−0.913	−1.384	−0.988	−1.027	−1.065	−0.596	−0.493	−0.080	0.247	−0.810	−1.330
Std. Error of Kurtosis	0.628	0.759	0.628	0.759	0.628	0.759	0.628	0.759	0.628	0.759	0.628	0.759	0.628	0.759	0.628	0.759	0.628	0.759	0.628	0.759
Minimum	0.000	0.060	0.020	0.043	0.050	0.050	12.000	12.000	6.000	5.000	18.000	18.000	7.000	9.000	8.000	8.000	15.000	13.000	14.000	29.000
Maximum	0.280	0.300	0.289	0.297	0.500	0.450	42.000	40.000	52.000	49.000	65.000	65.000	34.000	34.000	20.000	20.000	42.000	40.000	37.000	70.000
25th percentile	0.074	0.120	0.079	0.104	0.160	0.160	20.000	21.000	14.500	19.000	23.750	32.000	11.750	14.000	8.000	8.000	19.000	19.000	18.750	41.000
50th percentile	0.130	0.170	0.119	0.174	0.235	0.220	24.000	28.000	24.000	27.000	34.500	44.000	18.000	19.000	12.000	12.000	22.500	22.000	24.000	54.000
75th percentile	0.182	0.210	0.165	0.203	0.302	0.280	30.000	34.000	32.250	37.000	58.000	53.000	25.250	27.000	13.250	12.000	28.250	26.000	28.250	63.000

Note. Excluded 94 rows from the analysis that correspond to the missing values of the split-by variable group

Descriptive Statistics - Conclusion: Click here to add text
